# On the need for widespread horizontal gene transfers under genome size constraint

**DOI:** 10.1186/1745-6150-4-28

**Published:** 2009-08-25

**Authors:** Hervé Isambert, Richard R Stein

**Affiliations:** 1 Institut Curie, CNRS UMR168, 11 rue P. & M. Curie, 75005 Paris, France

## Abstract

**Background:**

While eukaryotes primarily evolve by duplication-divergence expansion (and reduction) of their own gene repertoire with only rare horizontal gene transfers, prokaryotes appear to evolve under both gene duplications and widespread horizontal gene transfers over long evolutionary time scales. But, the evolutionary origin of this striking difference in the importance of horizontal gene transfers remains by and large a mystery.

**Hypothesis:**

We propose that the abundance of horizontal gene transfers in free-living prokaryotes is a simple but necessary consequence of two opposite effects: *i) *their apparent genome size constraint compared to typical eukaryote genomes and *ii) *their underlying genome expansion dynamics through gene duplication-divergence evolution, as demonstrated by the presence of many tandem and block repeated genes. In principle, this combination of genome size constraint and underlying duplication expansion should lead to a coalescent-like process with extensive turnover of functional genes. This would, however, imply the unlikely, systematic reinvention of functions from discarded genes within independent phylogenetic lineages. Instead, we propose that the long-term evolutionary adaptation of free-living prokaryotes must have resulted in the emergence of efficient non-phylogenetic pathways to circumvent gene loss.

**Implications:**

This need for widespread horizontal gene transfers due to genome size constraint implies, in particular, that prokaryotes must remain under strong selection pressure in order to maintain the long-term evolutionary adaptation of their "mutualized" gene pool, beyond the inevitable turnover of individual prokaryote species. By contrast, the absence of genome size constraint for typical eukaryotes has presumably relaxed their need for widespread horizontal gene transfers and strong selection pressure. Yet, the resulting loss of genetic functions, due to weak selection pressure and inefficient gene recovery mechanisms, must have ultimately favored the emergence of more complex life styles and ecological integration of many eukaryotes.

**Reviewers:**

This article was reviewed by Pierre Pontarotti, Eugene V Koonin and Sergei Maslov.

## Background

With nearly 1,000 fully sequenced genomes, to date, and many more at a draft stage, comparative genomics has already highlighted major differences in the evolution of prokaryote and eukaryote genomes. In particular, a long and to some extent still ongoing debate [1-3] has helped delineate some quantitative differences in the amount of horizontal gene transfer across typical prokaryote and eukaryote genomes.

While interesting examples of horizontal gene transfers have been reported for a number of eukaryotes [4], these individually transferred genes of non-organelle origin amount to at most a few percent of the total number of genes in typical eukaryotic genomes (from virtually none in human [5] and <1% in rotifera [6] genomes up to 4% in ciliates from the rumen gut rich in bacteria-protist interactions [7]). Hence, following their likely arising from early symbiotic fusion between ancient archaebacteria and *α*-proteobacteria (with subsequent engulfment of cyanobacteria leading to plastids in plants) [8], eukaryotes appear to have, since then, primarily evolved by expansion (and reduction) of their ancestral gene repertoires through vertical inheritance of gene duplication-divergence events.

By contrast, prokaryotes rely on a seemingly more flexible evolutionary dynamics allowing for both rampant horizontal gene transfers between closely related species, as well as less frequent but evolutionary important gene transfers between phylogenetically distant species [9], such as between archaea and eubacteria [10-12]. All in all, it appears that only a small fraction of prokaryote genes are actually consistent with a universal phylogenetic tree a life [13], thereby suggesting that most prokaryote genes are eventually exchanged over long evolutionary time scales [1].

Yet, the evolutionary origin of these striking abundance of horizontal gene transfers in free-living prokaryotes remains by and large a mystery.

From a functional perspective, there is no doubt that some horizontally transferred genes do provide evolutionary benefit to their recipient host [14]. However, many transferred genes between prokaryotes appear to be evolutionary neutral or even deleterious as judged from their rapid turnover dynamics in typical prokaryote genomes [15-17]. So what is the evolutionary incentive for free-living prokaryotes to exchange many genes? Do they simply do it "because they can", having no separation of germline and soma, and possessing specific pumps for DNA intake, essentially, as food not "for the purpose" of gene transfer? (see E.V. Koonin's review below).

In this paper, we will argue that prokaryotes experience an abundance of gene transfers, not just because they can, but because they *have to*, owing to an inherent evolutionary *constraint*, specific to prokaryotes and absent for eukaryotes.

## Presentation of the hypothesis

**Figure 1:**
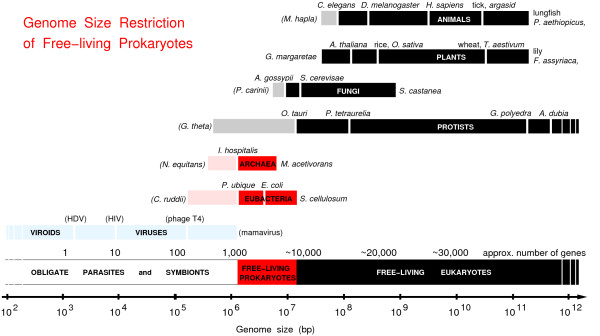
**Genome size restriction of free-living prokaryotes**. The genomes of free-living prokaryotes (archaea and eubacteria, in red) appear to be restricted to a mere 10-fold range in size from 1.3 Mbp to 13 Mbp, while the sizes of free-living eukaryote genomes (in black) span almost 10^5 ^folds from 10 Mbp to near 10^6 ^Mbp. The lower ranges of obligate parasite or symbiont genomes, shown as light pink and grey bars, can be much smaller than free-living prokaryote and eukaryote genomes, respectively, due to the progressive loss of dispensable genes. Viruses and gene-free viroids (in light blue) further reduce the size of parasitic "genomes" down to a few hundred nucleotides only.

We propose that the abundance of horizontal gene transfers in free-living prokaryotes is a simple but necessary consequence of two opposite effects: *i) *their apparent genome size constraint (Fig. 1) and *ii) *the underlying expansion dynamics of their genome through gene duplication-divergence evolution [18,19] (as well as amplification of short mobile elements in many free-living prokaryote genomes [19-21]).

In essence, the rationale of the proposed hypothesis is reminiscent of the situation of a population of organisms reproducing under global population size constraint. This is well known to lead to the inevitable turnover of genotypes in the population, if only through neutral drift. Yet, in the case of prokaryote genes under duplication-divergence evolution and genome size constraint, we will argue that the outcome should be quite different and lead to the evolutionary need for widespread horizontal gene transfers between prokaryotes, in place of an independent turnover of genes within different prokaryote genomes (as could be expected from a complete analogy with the results from population genetics).

Indeed, for population-level dynamics, the turnover of genotypes arises as population size constraints prevent the accumulation of individual organisms under limited amounts of space and/or food. Similarly, at the level of entire ecosystems, there is not enough space nor resources in a given environment to accommodate both newly arising species and all ancient species, which leads to an inevitable turnover of species, as long as the underlying speciation dynamics from extant species continues.

But is there a similar evolutionary restriction on the duplication-divergence expansion of gene repertoires due to an inherent genome size constraint for free-living organisms?

For eukaryotes, there is no indication that such a size constraint has yet been reached for typical genomes, which can reach vastly different sizes spanning more than 10,000 folds in length for free-living protists only, Fig. 1. So, there is no need to replace old genes by new ones due to size limitation in typical eukaryote genomes, which generally accommodate a large fraction of "junk" DNA relics as well.

By contrast, the genomes of free-living prokaryotes appear to be limited to a 10-fold variation in size, from about 1.3 Mbp to 13 Mbp (Fig. 1), with proportionally very limited space for retaining non-functional DNA (*i.e*. a few percent of their genome at most), especially in free-living prokaryotes with large genomes (>7 Mbp) [19,20].

Note, moreover, that the compactness of prokaryotic genomes is not the cause of their narrow range of genome sizes. Indeed, obligate parasites and symbionts, which also typically present compact "genomes", can span a much wider range of genome sizes with essentially no lower limit in gene content for the extreme parasitic lifestyles of many viruses and viroids.

So, what is the origin of the narrow range of genome sizes for free-living prokaryotes? While the lower limit of about 1 Mbp likely corresponds to a putative minimal genome for free-living lifestyle, the origin of the upper genome limit of about 10 Mbp (Fig. 1) is more speculative at this stage.

Although selection pressure for genome streamlining [22] might be an important evolutionary drive for some free-living prokaryotes as well as eukaryotes with large population sizes (as for the abundant maritime bacteria, *Pelagibacter ubique *[23] or green algeae, *Ostreococcus tauri *[24]), it does not appear to be a general trend amongst free-living prokaryotes [20], which even show a significant anticorrelation between genome size and selection pressure [3].

This suggests that the apparent genome size restriction of free-living prokaryotes, Fig. 1, is not caused by an adaptive streamlining of their genomes but may, instead, result from an inherent evolutionary constraint acting to limit their genome expansion. Such a genome size restriction could, for instance, stem from a tight constraint on the surface to volume ratio of free-living prokaryotes, due to their need to directly extract energy and food from their surrounding environment. By contrast, eukaryotes have specialized intracellular organelles to fulfill these tasks, such as mitochondria and plastids, whose number can be scaled in proportion to the intracellular needs of eukaryotic cells [25]. Note, in particular, that the observed 10-fold restriction in genome size (*L*) of free-living prokaryotes (Fig. 1) actually corresponds to a tighter 2-fold limitation on their surface to volume ratio *S/V ~ L*^-1/3 ^(assuming *L ~ V ~ S*^3/2^). But the apparent genome size restriction might possibly stem from other evolutionary constraints of free-living prokaryotes, such as a cell size limit for efficient intra-cellular diffusion of metabolites and proteins.

Alternatively, genome size constraints might also result from a more operational limitation of prokaryote functional regulation, with a possible "bureaucracy ceiling" [3,26] or "microeconomic" optimisation principles [27] that have been proposed for prokaryote regulatory systems. Yet, considering the virtually unlimited possibility and complexity of regulatory systems, such operational limitations seem more likely to be themselves the result of built-in physical constraints or elementary principles of evolution than to be the primary force behind tight genome size restriction of free-living prokaryotes (see, Testing of the hypothesis).

It remains that, whatever the actual origin of such genome size constraint, it should oppose the evolutionary expansion of prokaryote genomes. Yet, direct evidences of gene duplication-divergence dynamics and genome expansion are provided by the significant proportion of tandem or block repeated genes (*e.g*. 15-25%) [18,19] and the presence of short mobile elements (*e.g*. 5%) [19-21], in particular for medium sized genomes (3-7 Mbp), whereas the proportion of short mobile elements is typically somewhat smaller (*e.g*. < 1-2%) in large prokaryote genomes (>7 Mbp) [19,20]. This is consistent with the underlying notion of an increasing "gene pressure" as prokaryote genomes get closer to an effective upper size limit.

In principle, pervasive gene duplication dynamics should then lead to a continuous turnover of genes in different free-living prokaryote species, by analogy with the well-known coalescent process from population genetics, as outlined above. However, long-term evolutionary adaptation of living organisms could not rely on such a continual reinvention of functions from discarded genes, as it would essentially consist in achieving adaptation through systematic gene displacement, without the possibility to learn from previous successful evolutionary pathways [28,29]. Instead, the long-term evolutionary adaptation of living organisms is well known to mainly rely on tinkering with the long evolved functions of extant genes and their regulations. Hence, by precluding long-term phylogenetic inheritance of functional genes within independent prokaryote lineages, genome size constraints must have favored the necessary emergence of alternative ways to recover lost genes from other prokaryote genomes or possibly other genetic reservoirs, such as bacteriophage viruses. In other words, genes that are non-essential in a given environment and, hence, likely to be lost under genome size constraint, must be recoverable from other free-living or parasitic species to avoid an otherwise irreversible decline in gene content and thereby adaptive potential of *all *prokaryote lineages. Instead, widespread use of horizontal gene transfer results in the long-term evolutionary adaptation of a "mutualized" gene pool [3], that is maintained and diversified through and beyond the turnover of individual prokaryote species.

## Testing of the hypothesis

The proposed hypothesis, linking genome size constraint and gene duplication-divergence dynamics to widespread horizontal transfers in free-living prokaryotes, is broadly supported by the comparative genomic analysis highlighted above.

Ideally, this hypothesis might also be directly testable experimentally, using genome engineering approaches [30], although designing and interpreting an experiment to probe the evolution of the size constraint of a bacterial genome might not be an easy task.

Alternatively, it would also be interesting to test, on theoretical grounds, whether the interplay between genome size constraints and gene duplication-divergence evolution could account for other seemingly unrelated features of free-living prokaryotes.

In particular, the opposite effects of gene duplication-divergence evolution and genome size constraint, leading to widespread horizontal gene transfers, are unlikely to apply uniformly over the whole collection of prokaryote genes, which perform a wide range of distinct cellular functions. This is indeed consistent with the available data showing that prokaryote genes are subjected to different rates of horizontal gene transfer, reflecting at least in part their different cellular functions [31]. Yet, most genes do appear to be eventually transferred between prokaryote species, over long evolutionary time scales, except for a central core of possibly a few hundred vertically inherited genes [1,9].

So, the same genome size constraint likely leads to different rates of horizontal gene transfer for different types of genes. This should in turn affects their relative abundances and possible interactions in functional biological networks involving *different *gene types, such as transcription networks or signal transduction networks [32]. In fact, such oriented networks, between regulators and target genes or between enzymes and substrate genes, are known to exhibit gene type-specific expansions depending on the actual size of their genome [26]. These oriented networks also present distinct global topologies in prokaryotes and eukaryotes [32-34].

Besides, the fact that inherent evolutionary constraints can have farranging implications on the emerging properties of biological systems is not new. In particular, population size (*N*) constraints have long been known to restrict the effective range of adaptive selection to high fitness gains, *s *> 1/*N*, while favoring random evolutionary drift in place of lower fitness gains, *s *< 1/*N*. But, beyond population-level constraints, there are also inherent evolutionary constraints at the level of individual genomes, which ultimately restrict, by construction, the possible evolution of living organisms.

For instance, we have demonstrated [28,29] that, in *absence *of genome size constraint (which presumably applies to typical eukaryote genomes, see below), duplication-divergence processes already entail by themselves strong restrictions on the emerging molecular organization of cellular functions. In particular, duplication-divergence evolution directly restricts, by construction, the emerging structure of conserved biomolecular networks to scale-free topologies, irrespective of any biological function [28].

Concerning more directly the topology of prokaryote molecular networks, Maslov *et al*. [35] have recently proposed an interesting "toolbox model" accounting for the topology and evolution of their metabolic networks. It assumes that one transcription factor and a minimal number of metabolic enzymes are transferred on-demand when needed from a "home depot" of metabolic genes (see S. Maslov's review below).

These examples illustrate how the topologies of biological networks might indeed be related to simple and seemingly unrelated evolutionary processes such as elementary duplication-divergence processes [28,29] or on-demand horizontal gene transfers [35].

Similarly, we expect that the evolutionary consequences of genome size constraint on free-living prokaryotes (Fig. 1) are bound to extend from the mere restrictions on genome evolution, such as the need for widespread horizontal gene transfer discussed here, to more integrated operational constraints at the level of cellular functions and regulations.

## Implications of the hypothesis

The main implication of this need for widespread horizontal gene transfers under genome size constraint is the emergence of a "mutualized" gene pool, as outlined above. Indeed, by "mutualizing" a pool of exchangeable genes, widespread use of horizontal gene transfers circumvents the otherwise inevitable loss of many genes in each prokaryote lineages.

Yet, the long-term evolutionary adaptation of such a mutualized gene pool, beyond the inevitable turnover of prokaryote species, requires that free-living prokaryotes remain under strong selection pressure. Indeed, widespread random horizontal gene transfers under weak selection pressure could only be deleterious, in the end, for the gene pool and its prokaryote hosts. Hence, on long evolutionary time scales, only genes that provide some sort of advantage to their prokaryote hosts in specific environments are expected to be preserved in the mutualized gene pool.

Conversely, by freeing themselves from genome size constraints, typical eukaryotes can actually conserve long evolved genes in their own genome under much weaker selection pressure and smaller population sizes than prokaryotes. This sets the stage for a radically different exploration of the genotype-phenotype space of eukaryotes [36], as compared to the adaptation-driven selection of prokaryotes and their "mutualized" gene pool.

Indeed, from a global evolutionary perspective, different eukaryote lineages appear to be exploring, in "parallel", various combinatorial expressions of conserved genes, evolving under near neutral genomic duplication-divergence dynamics and random speciation events [28,36]. As a result, most of these eukaryote lineages are likely to die out on the way, in agreement with the typical eukaryote species life span of about 1 to 10 million years only. Yet, we expect that non-adaptive evolution of eukaryotes can be globally sustained as long as successful lineages, escaping background extinction and occasional mass extinction events, continue to provide enough eukaryote diversity through further speciation events.

But how such parallel evolutionary dynamics from conserved genes can lead to the great diversity of known eukaryotes under weak selection pressure?

On short evolutionary time scales, the diversity of eukaryotes is thought to be driven by independent changes in gene regulation and occasional expansions of gene families. This presumably underlies the striking ability of higher eukaryotes to "adapt", apparently by chance, to diverse natural environments when the opportunity arises. It is illustrated, for instance, by the three independent returns to aquatic life of pinnipeds, cetaceans and sirenians, some 30 to 50 MY ago, from three different lineages of terrestrial mammals under possible change in feeding ecology [37]. Similarly, the return of traits lost in distant ancestors is supported by an increasing number of reports suggesting that stick insects can regain wings, lizards can regain digits, slipper limpets can regain a coiled shell, asexual mites can regain sex, frogs can regain tadpoles in their life histories and marine snails can regain a feeding larval stage (see [38] for review). This ability of eukaryotes to maintain the potential to produce traits lost in distant ancestors typically results from cis-regulatory changes in the control of genes [39] that have always remained functional, but in other genomic contexts, *e.g*. different development stages or differentiated tissue types. By contrast, the resurrection of pseudogenes remains seemingly exceptional, due to the accumulation of errors in sequence coding regions beyond 5-10 MY [38], although a few examples have recently been reported [40] (see P. Pontarotti's review below).

But, beyond changes in gene regulation and occasional resurrection of pseudogenes, the actual functions of orthologous genes can also diverge, and will eventually do so, across phylogenetic distant lineages. This is due to the inevitable turnover of interaction partners of orthologous genes under duplication-divergence dynamics over long evolutionary time scales (*e.g*. > 100-200 MY), as demonstrated in [28], Fig. 5.

Finally, on even longer evolutionary time scales (*e.g*. >500 MY), weak selection pressure, population bottlenecks and inefficient horizontal gene transfers inevitably lead to the accidental loss of temporarily dispensable genes as, for instance, from rarely used metabolic pathways. This has presumably contributed to the emergence of more complex life styles and ecological integration for many eukaryotes, which must ultimately rely on external sources for essential metabolite intermediates, such as certain amino acids, they can no longer produce themselves.

In summary, the need for widespread horizontal gene transfers, due to size restriction of prokaryote genomes, has likely favored the emergence and progressive adaptation of a mutualized gene pool with increasingly elaborate functions, in spite of the inevitable turnover of individual prokaryote species. By contrast, the absence of genome size constraint for typical eukaryotes has relaxed the need for widespread horizontal gene transfers and strong selection pressure, which presumably governed the evolution of their pre-symbiosis prokaryote ancestors. But the inevitable loss of genetic functions, under weak selection pressure and inefficient gene recovery mechanisms, must have favored the emergence and ultimate success of more complex life styles and ecological integration of many eukaryotes.

## Competing interests

The authors declare that they have no competing interests.

## Authors' contributions

HI designed research and wrote the paper. HI and RS performed research.

## Reviewers' comments

Reviewer 1: Dr Pierre Pontarotti, Evolution Génome Environnement, Université d'Aix Marseille, Marseille, France

The authors propose that the genome size constraint and the genome evolution process of archea* and bacteria* (gene duplication: divergence) lead to gene function loss and that these losses need to be compensated by genes arising from other species. A process called Horizontal Gene Transfer (HGT).

In the case of eukaryotes, the genome size does not seem to be a problem; therefore the genes that are not used can be conserved during a long time period and could still be re-used after a while. If this time is too long then, the genes will be lost but gene function resurrection (or back/reverse cooption) could occur.

Comments:

1) To strengthen their hypothesis, the authors need to give some examples of resurrected gene function in eukaryotes.

Authors' response

*A recently reported example of resurrected gene in eukaryotes is an immunity-related GTPase (IRG) gene in humans, IRGM, which was shown *[40]*to have resurrected about 20 MY ago in all human and great ape lineages after staying dormant as pseudogenes for about 25-30 MY. But such resurrections of pseudogenes remain seemingly exceptional beyond 5-10 MY, due to the accumulation of errors in sequence coding regions *[38].

*By contrast, the return of traits lost in distant ancestors, which is supported by an increasing number of reports (see *[38]*for review), does not typically involve the resurrection of pseudogenes. Instead, it usually corresponds to cis-regulatory changes in the control of genes *[39]*that have always remained functional, but in other genomic contexts*, e.g. *different development stages or differentiated tissue types. This is an important point, which we have now underlined more clearly*.

2) At the end of the paper, the authors give an example of style life returns, several other examples are found on character return that seem to me more appropriate. Clear example of reverse evolution for a given character could be found in the following references:

a) Evidence for the reversibility of digit loss: A phylogenetic study of limb evolution in Bachia Gymnophthalmidae: Squamata, Kohlsdorf T and Wagner. GP EVOLUTION 60: 9 Pages: 1896-1912, 2006.

And b) Limpets break Dollo's law Pagel M: TRENDS IN ECOLOGY & EVOLUTION 19:6 Pages: 278-280, 2004.

Authors' response

*We thank Dr Pierre Pontarotti for pointing to us these relevant and interesting papers *[41,42]*which illustrate the ability of eukaryotes to maintain the potential to produce traits lost in distant ancestors*.

3) Do the authors look at eukaryotic phyla in which HGT occurred and do they found an inverse correlation between the level of HGT and the genome size (or with genes number ...)?

Authors' response

*We have not looked at eukaryotic phyla with HGT in details, but the evidences of HGT that have been reported for eukaryotes seem more directly related to their promiscuous life style with bacteria in bacteria-rich environments and/or the relative accessibility of their germline to HGT*.

4) Minor comments The part of the article: testing of the hypothesis should be re-written as it is very hard to follow (even if we read the previous article published by the authors. Please consider revision of this section to clarify.

Authors' response

*We have in fact significantly simplified the discussion related to refs *[28,29], *which could not be exposed in sufficient details in the format of an hypothesis paper*.

*I think that the word prokaryote is misleading

**Reviewer 2: Dr Eugene V Koonin, National Center for Biotechnology Information, NIH, Bethesda, Maryland, United States**.

This is an interesting Hypothesis paper that interprets the pervasive horizontal gene transfer (HGT) in prokaryotes as a "simple but necessary consequence" of their apparent genome size constraints and the "underlying expansion dynamics of their genome through gene duplication-divergence evolution". In itself, this is a straightforward, sound, and yet, interesting idea. To my knowledge, this point has never been explicitly discussed before which I find surprising. Indeed, if there is a tight size constraint, and at the same time, a characteristic rate of gene duplication, there also should be some force to maintain and restore functional diversity, especially, in the context of a community genome, and HGT is the best and obvious candidate. I think theoretical work that shows the possibility of long-time persistence of genes acquired via HGT, even in the absence of measurable selective advantage is relevant here:

Novozhilov et al. Mathematical modeling of evolution of horizontally transferred genes. MBE 2005; 22: 1721-1732 Moreover, I suspect that the hypothesis discussed in this paper in itself provides for fairly straightforward mathematical modeling - perhaps, not for this paper but I think it would be interesting to do.

Authors' response

*We thank Dr Eugene V Koonin for his insightful expertise and for pointing out Novozhilov et al.'s paper *[15]*to us. We agree that the hypothesis we propose provides in itself for fairly straightforward mathematical modeling. Yet, our main intent in this "hypothesis" paper is precisely to put the emphasis on the *premises *of the argument (*i.e. *genome size constraints and duplication-divergence evolution) rather than on a specific mathematical model illustrating the resulting need for widespread HGT*.

This being said, I am not sure that I find the section on "testing of the hypothesis" particularly illuminating or even genuinely relevant. The connection between network topology and HGT escapes me. At the very least, it would be helpful to explain this in more explicit terms. I also find the section on implications of the hypothesis rather vague and do not believe that the excursion into the raisond' etre of eukaryotes is particularly helpful. A more careful and concrete discussion of the evolution of prokaryotes themselves would do more for the exposition of the authors' hypothesis.

Authors' response

*We have clarified the sections about "testing" and "implication" of the hypothesis. Ideally, hypothesis should be directly tested by experiments, yet designing dedicated experiments on genome evolution is typically not an easy task. Alternatively, one can either look for counter-examples, which would invalidate the hypothesis, or demonstrate that the proposal carries in fact further, less direct consequences, that are also consistent with additional empirical data. This is the sort of "tests", we would like bring forward in further follow up studies. While the connection between network topology and HGT might not seem so direct at first sight, it is nonetheless expected, we believe, insofar as HGT do affect the gene repertoire and hence the molecular interactions within biological networks. This is well illustrated for example in the "Toolbox model of evolution of prokaryotic metabolic networks and their regulation" by Maslov et al*. [35], *see below*.

My further misgivings about this and similar papers are not even criticisms but more philosophical musings about the status of "Why?" questions in biology. This paper tries to address that very sort of question: why so much HGT among prokaryotes? Answers can be given at a number of different levels, and Isambert and Stein offer one of them, a very interesting one, rooted in constraints and features of genome evolution. But one can also easily argue that prokaryotes, basically, do it so often because they can, having no separation of germline and soma, and possessing specific pumps for DNA intake, essentially, as food not "for the purpose" of HGT.

Authors' response

*The fact that prokaryotes have no separation of germline and soma and possess specific pumps for DNA intake likely facilitates the horizontal transfer of genes in their genomes. Yet, many unicellular eukaryotes, without separation of germline and soma and also commonly exposed to foreign DNA (*e.g. *through feeding on bacteria), happen to experience much less HGT (<1%) than typical prokaryotes. Hence, the absence of germline and soma separation together with regular uptake of exogenous DNA are not sufficient conditions to account for widespread HGT nor are they, in fact, necessary conditions, as ciliates from the rumen gut appear to have experienced a relative abundance of HGT *[7]*(about 4% of their genes), despite their separate germline micronucleus and somatic macronucleus*.

*As for the status of "why?" questions in biology, we entertain the idea that it should be exactly the same as in any other scientific field. Hence, the premise is that many observations on biological systems are not independent from one another and may in fact be "explained", that is, logically related to one another, if only at a statistical level. While we agree that "answers can be given at a number of different levels", it is also clear that different answers might not provide the same level of "understanding". For instance, it can be argued that the proposition that "prokaryotes experience an abundance of HGT because they have to" is a "stronger" (that is, more constrained) hypothesis than "prokaryotes, basically, do [HGT] so often because they can", which should also apply, in principle, to unicellular eukaryotes without separation of germline and soma and commonly exposed to foreign DNA, as discussed above*.

More broadly, one could argue that HGT, or put another way, mixing and matching of genetic elements is the primary mode of life existence that does not call for an explanation (this is, in a slightly caricatured form, the view propounded in ref. [3]) whereas everything that deviates from that modality, certainly, eukaryotes, but to some extent, any cellular life forms, needs to be explained. This is not so much criticism of the present paper but rather a series of general thoughts on the epistemology of evolutionary biology. Along these lines, the paper is not really a Hypothesis, at least, not in the strict Popperian sense, but rather a viewpoint. This somewhat skeptical position that I take does not render the paper uninteresting or useless.

Authors' response

*We believe that we present a genuine hypothesis which does not concern the existence of any primitive form of life but merely the consequence of the apparent genome size constraint and underlying duplication-divergence evolution of free-living prokaryotes, as we know them. In particular, we would like to stress that the abundance of HGT cannot in itself restrain the size of prokaryote genomes (nor does it imply duplication-divergence evolution of their genomes). So, deriving the abundance of HGT among free-living prokaryotes from their apparent genome size constraint and underlying duplication-divergence evolution is not a circular argument. It is, we believe, a genuine hypothesis, which could in principle be falsified. As pointed out in the paper, this would involve to find a free-living prokaryote lineage having achieved long-term adaptation through systematic gene displacements rather than gene transfers. Although at odd with known evolutionary trends, one cannot exclude a priori the existence of such isolated free-living prokaryotes, which would need to continually reinvent the functions of discarded genes without long-term memory of previous evolutionary successes*.

Minor comment:

p. 3, left: I suggest not lumping viruses and viroids with prokaryotes when considering genome size constraints. I would limit this discussion to cellular life forms in which case a lower limit does seem to exist, although the discovery of tiny endosymbionts like Carsonella pushes this limit surprisingly low.

Authors' response

*We have cut this paragraph in two to avoid any confusion*.

**Reviewer 3: Dr Sergei Maslov, Brookhaven National Laboratory, Upton, NY, United States**.

The manuscript contains a speculative argument that widespread horizontal gene transfer in prokaryotes is an adaptation allowing them to preserve long-term evolutionary memory that would otherwise have been quickly erased by rapid gene turnover.

This view goes along with my recent "Home Depot" model of prokaryotic evolution (S. Maslov, S. Krishna, T. Y. Pang, and K. Sneppen, "Toolbox model of evolution of prokaryotic metabolic networks and their regulation", in press (2009)). In our model prokaryotic genomes are constantly replenished from a common repository with entire metabolic pathways. This process can be compared to constantly buying tools in a hardware store (hence the "Home Depot" metaphor) only to return them once the project is over.

Authors' response

*We thank Dr Sergei Maslov for mentioning to us this interesting paper on the "Toolbox model of evolution of prokaryotic metabolic networks and their regulation" *[35]. *In particular, we would like to point out that, beyond the pivotal role of HGT (which we argue must be widespread under genome size constraint), the premise of the "toolbox model" also appears to rely on an additional finite size constraint of prokaryote evolution, namely, the apparent finite size of their available metabolic universe, N*_univ_. *Based on current KEGG data, Maslov et al. postulate that there are about N*_univ _= 1,800 *metabolic compounds that can be metabolized by specific enzymes, which prokaryotes can alternatively acquire or discard through HGT. Then, the toolbox model predicts that the number of new metabolic enzymes that can be acquired and controlled by a single new transcription factor eventually decreases as the size of their metabolic network N*met *approaches N*_univ_. *Hence, it follows that the interplay between duplication-divergence evolutionary dynamics of prokaryotes under genome size constraints and the finite size of their available metabolic environment might ultimately control not only their requirement for widespread HGT but also the global topology of their metabolic networks*.

From my standpoint, this manuscript would greatly benefit if authors would accompany their verbal argument with a quantitative model. The model does not have to be realistic it just needs to clearly make the main point of the argument that prokaryotic world would collapse without some sort of gene exchange with a mutualized gene pool. Do authors expect a sharp error-catastrophe-like phase transition *in the absence* of horizontal gene transfer? What numerical constant is best suited to quantify this transition (akin to the number of mutations per genome per generation for error catastrophe)? What is its (approximate) range in prokaryotes? In the section "Testing the hypothesis" authors repeatedly refer to their earlier work (Ref. [27]) without adequately explaining even the basic ingredients of this earlier model. This section needs to be significantly expanded in the revised version of the manuscript.

Authors' response

*After considering to significantly expand the section on "Testing the hypothesis", we have eventually opted out to greatly simplify the discussion related to refs *[28,29]. *Indeed, we felt that the full model developed in *[28,29]*could not be adequately presented and further analyzed within the intended format of an hypothesis paper*.

*Similarly, we agree that a simple "error-catastrophe-like model" could be worked out in the case of gene-type independent genomes, but such an homogenous gene-type model could hardly been seen as realistic, as pointed out by S. Maslov. Gene-type dependent HGT should undoubtedly be taken into account as discussed in the section on "Testing the hypothesis"*.

*Again, our main intent in this "hypothesis" paper is to put the emphasis on the *premises *of the argument (*i.e. *genome size constraints and duplication-divergence evolution) rather than on a specific mathematical model illustrating the resulting need for widespread HGT*.
